# Inhibitory effect of *Porphyromonas gingivalis*‐derived phosphoethanolamine dihydroceramide on acid ceramidase expression in oral squamous cells

**DOI:** 10.1111/jcmm.17722

**Published:** 2023-04-05

**Authors:** Chiaki Yamada, Anny Ho, Amilia Nusbaum, Ruijuan Xu, Mary Ellen Davey, Frank Nichols, Cungui Mao, Alexandru Movila

**Affiliations:** ^1^ Department of Biomedical Sciences and Comprehensive Care Indiana University School of Dentistry Indianapolis Indiana USA; ^2^ Indiana Center for Musculoskeletal Health Indiana University School of Medicine Indianapolis Indiana USA; ^3^ Institute for Neuro‐Immune Medicine Nova Southeastern University Fort Lauderdale Florida USA; ^4^ Department of Medicine, Stony Brook Cancer Center Renaissance School of Medicine The State University of New York at Stony Brook Stony Brook New York USA; ^5^ Department of Microbiology The Forsyth Institute Cambridge Massachusetts USA; ^6^ Department of Oral Health and Diagnostic Sciences University of Connecticut School of Dental Medicine Farmington Connecticut USA

**Keywords:** ceramide, oral cavity bacteria, oral squamous cell carcinoma, phosphoethanolamine dihydroceramide, *Porphyromonas gingivalis*

## Abstract

The maintenance of diminished acid ceramidase (*ASAH1*) gene expression leading to the accumulation of antiproliferative intracellular ceramides in oral squamous cell carcinoma (OSCC) has emerged as a prospective oral cancer therapeutic regimen. Our published study demonstrated that the key periodontal pathogen *Porphyromonas gingivalis* downregulates the expression patterns of *ASAH1* mRNA in normal epithelial cells in vitro*.* Therefore, *P. gingivalis* may also beneficially diminish the expression of *ASAH1* in OSCC. Because a uniquely structured *P. gingivalis*‐derived phosphoethanolamine dihydroceramide (PEDHC) inhibits the proliferation of normal human fibroblasts, this study aimed to test the effect of PEDHC on the survival of human oral squamous OECM‐1 cells in vitro. We demonstrated that the *P. gingivalis* dihydroceramide‐null (ΔPG1780) strain upregulates the expression of ASAH1 mRNA and promotes aggressive proliferation and migration of OECM‐1 cells compared to the parent *P. gingivalis*‐W83 strain. In addition, the intracellular concentration of ceramides was dramatically elevated in OECM‐1 cells exposed to PEDHC in vitro. Furthermore, PEDHC inhibited expression patterns of *ASAH1 mRNA* as well as some genes associated with degradation of the basement membranes and extracellular matrix, for example, MMP‐2, ADAM‐17 and IL‐6, in OECM‐1 cells. Altogether, these data indicated that PEDHC produced by *P. gingivalis* inhibits acid ceramidase expression, promotes intracellular ceramide accumulation and suppresses the survival and migration of OSCC cells in vitro. Further studies are needed to determine molecular mechanisms of PEDHC‐mediated inhibitory effect(s) on OSCC using in vivo models of oral cancer.

## 1 INTRODUCTION

Oral squamous cell carcinoma (OSCC) is one of the most common malignant cancers of the head and neck and is among the top 10 common cancers worldwide.^1^ Notably, OSCC can alter sphingolipid metabolism towards increasing proliferative species such as sphingosine‐1‐phosphate (S1P) while decreasing antiproliferative species such as ceramide. Furthermore, the ceramide/S1P ratio is regulated by acid ceramidase (*ASAH1*).^2^, ^3^ Therefore, the maintenance of diminished *ASAH1* gene expression leading to the accumulation of intracellular ceramide in OSCC has emerged as a potential objective for oral cancer therapy.

We previously reported that infection with oral bacteria *Porphyromonas gingivalis* downregulates the expression of *ASAH1* mRNA in normal epithelial cells, thus promoting inflammation and cell apoptosis associated with periodontal disease.^4^ Emerging evidence demonstrated that a uniquely structured phosphoethanolamine dihydroceramide (PEDHC), produced by microbes from *Bacteroides* spp., including *P. gingivalis*, contributes to intracellular ceramide accumulation in normal epithelial tissue.^5^, ^6^ While the association of *P. gingivalis* with oral and neck cancer pathology was intensively studied,^7^, ^8^ the effects of *P. gingivalis*‐derived PEDHC on the OSCC remains unclear.

In this study, we investigated the impact of *P. gingivalis*‐W83, W83‐dihydroceramide null mutant strain (ΔPG1780) and PEDHC on the proliferation and migration of OSCC in vitro. Here, we also addressed whether PEDHC affects intracellular ceramide metabolism in OSCC.

## 2 MATERIALS AND METHODS

Detailed materials and methods are provided in the Supplementary Material and Methods.

## 3 RESULTS AND DISCUSSION

**FIGURE 1:**
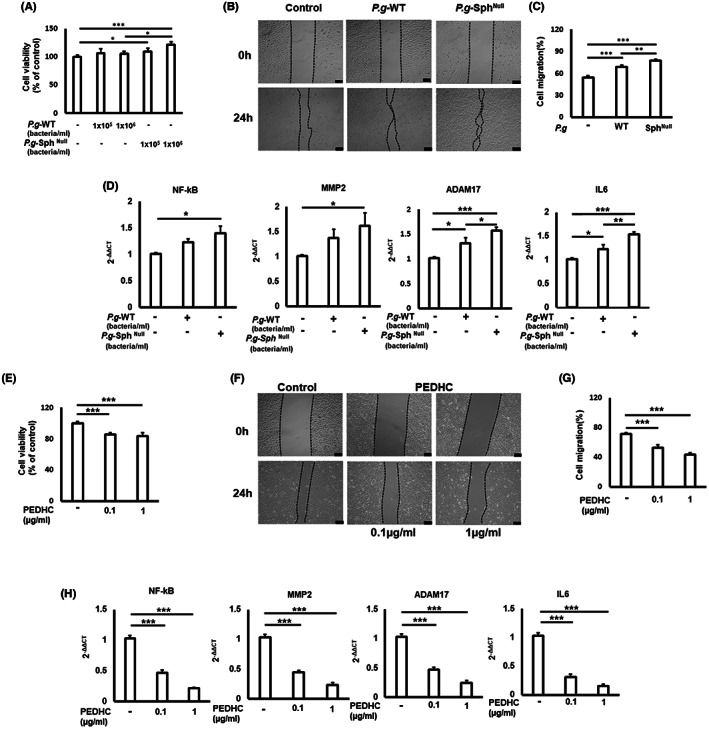
Effect of live *Porphyromonas gingivalis* (P.g) dihydroceramide sphingolipids null ΔPG1780 (*P.g*‐Sph^Null^), and *P. gingivalis*‐W83 wild‐type (P.g‐WT) control strains, and isolated PEDHC on the proliferation and migration of oral squamous OECM‐1. (A) Proliferation of oral squamous cancer OECM‐1 cells in the presence of P.g‐WT and *P.g*‐Sph^Null^ strains. (B) Representative images of scratched and recovered wounded areas (marked by black lines) on confluence monolayers of OECM‐1 cancer cells at different time points exposed to P.g‐WT and *P.g*‐Sph^Null^ strains. (C) A semi‐quantitative analysis of wound closure. (D) Expression of genes associated with degradation of the basement membrane and extracellular matrix, including NF‐kB, MMP2, ADAM17, and IL‐6 in OECM‐1 cells. (E) Proliferation of oral squamous cancer OECM‐1 cells in the presence of various concentrations of PEDHC solution in PBS or PBS alone. (F) Representative images of scratched and recovered wounded areas (marked by black lines) on confluence monolayers of OECM‐1 cancer cells at different time points exposed to PEDHC or control (no PEDHC) cells. (G) A semi‐quantitative analysis of wound closure. (H) Expression of genes associated with degradation of the basement membrane and extracellular matrix, including NF‐kB, MMP2, ADAM17, and IL‐6 in OECM‐1 cells. Data are shown from three independent experiments. *p < 0.05, **p < 0.01, ***p < 0.001.

Among the known clinically relevant *P. gingivalis* strains, the W83 strain contributes to severe periodontitis in various experimental mouse models.^9^, ^10^ Therefore, *P. gingivalis*‐W83 and ΔPG1780 were used to infect healthy human oral gingival OBA‐9 and squamous cell carcinoma OECM‐1 cells in vitro. To our knowledge, exposure of OBA‐9 cells to either *P. gingivalis*‐W83 or ΔPG1780 significantly inhibited their proliferation. In contrast, no or little effect of *P. gingivalis*‐W83 was observed on the proliferation of OECM‐1 cells, whereas exposure to ΔPG1780 increased the proliferation of OECM‐1 cells (Figure 1A). We also observed that exposure to live *P. gingivalis*‐W83 or ΔPG1780 significantly elevated migration of OECM‐1 cells compared to a non‐infected controls, with the mutant strain being more effective than the wild‐type strain (Figure 1B, C). Furthermore, ΔPG1780 significantly elevated the expression of *NF‐kB*, *MMP2*, *ADAM17* and *IL‐6* mRNAs in OECM‐1 compared to the parent *P. gingivalis*‐W83 strain (Figure 1D). These results suggest that *P. gingivalis‐*W83 inhibits proliferation and migration of OECM‐1 cells when it can synthesize dihydroceramide sphingolipids. However, a recently published systemic review highlighted the relationships between *P. gingivalis* ATCC33277 and 381 strains in OSCC development.^11^

Because our data indicated that the incubation with the ΔPG1780 strain promotes the proliferation of OECM‐1 oral squamous carcinoma cells (Figure 1), we next examined the impact of PEDHC, purified from *P. gingivalis*, on the proliferation of OECM‐1 cells in vitro. The viability of OECM‐1 cells was significantly reduced in the presence of PEDHC (Figure 1E). Furthermore, PEDHC also inhibited migration of OECM‐1 cells (Figure 1F, G) and expression patterns of *NF‐kB*, *MMP2*, *ADAM17* and *IL‐6* mRNAs in vitro (Figure 1H). In addition, PEDHC significantly increased concentrations of ceramides in OECM‐1 cells compared to the control group (Figure S, 1), indicating that PEDHC elevates the intracellular concentrations of ceramides in oral squamous carcinoma cells in vitro. It was reported that the decreased intracellular ceramide species positively correlate with an elevated proliferation of OSCC.^12^, ^13^

The critically low concentration of intracellular ceramide sphingolipids in oral squamous carcinoma could be caused by increased ceramidases activity which occupies a powerful position in the catabolism of pro‐apoptotic ceramide and generation of pro‐survival S1P bioactive lipid.^14^, ^15^, ^16^ Among the known ceramidases, the expression pattern of acid ceramidase (ASAH1) mRNA was dramatically elevated in OECM‐1 cells compared to OBA‐9 cells (Figure S2). Since we reported that only ASAH1 was inhibited in normal OBA‐9 cells by *P. gingivalis‐*ATCC3327 strain,^4^ we validated the effect(s) of the acid ceramidase chemical inhibitor LCL‐521 on the proliferation and migration of oral OECM‐1 squamous cells. As expected, LCL‐521 significantly reduced the expression of *ASAH1* mRNA in OECM1 cells in a concentration‐dependent manner (Figure S3A). Furthermore, expression of *NF‐kB*, *MMP2*, *ADAM17* and *IL6* genes were diminished in OECM‐1 cells exposed to LCL‐521 (Figure S3B). We also detected that LCL‐521 downregulated expression of *S1PR1*, and *S1PR3* mRNAs (Figure S3C). These S1PR1 and S1PR3 congenic receptors are significant in the S1P‐mediated survival and chemotaxis of cancer cells.^14^, ^17^ Finally, we also observed that the viability and migration of OECM‐1 were significantly inhibited by LCL‐521 compared to the sham control (Figure S3B–D).

Because our data indicated that acid ceramidase plays an essential role in the regulation of cancer cell viability, we postulated next that PEDHC affects OECM‐1 survival via downregulation of *ASAH1* mRNA. Exposure of OECM‐1 cells to the ΔPG1780 strain significantly elevated the expression of *ASAH1* mRNA when compared to *P. gingivalis‐W83*. Furthermore, we observed no significant effect of *P. gingivalis‐W83* on the *ASAH1* expression in OECM‐1 cells (Figure S4A). In addition, a concentration‐dependent downregulation of *ASAH1* was observed in OECM‐1 cells exposed to the PEDHC (Figure S4B). These data agree with earlier published observations indicating that acid ceramidase is critical in the survival of cancer stem cells in melanoma, glioblastoma and colon cancer.^2^, ^18^ Furthermore, it was also demonstrated that genetic and pharmacological inhibition of acid ceramidase prevents asymmetric cell division, which is frequently observed in cancer patients.^19^ Therefore, the maintenance of diminished acid ceramidase expression or activity leading to the accumulation of intracellular ceramide in OSCC and other types of cancer has emerged as a potential objective for cancer therapy.^20^, ^21^

**FIGURE 2:**
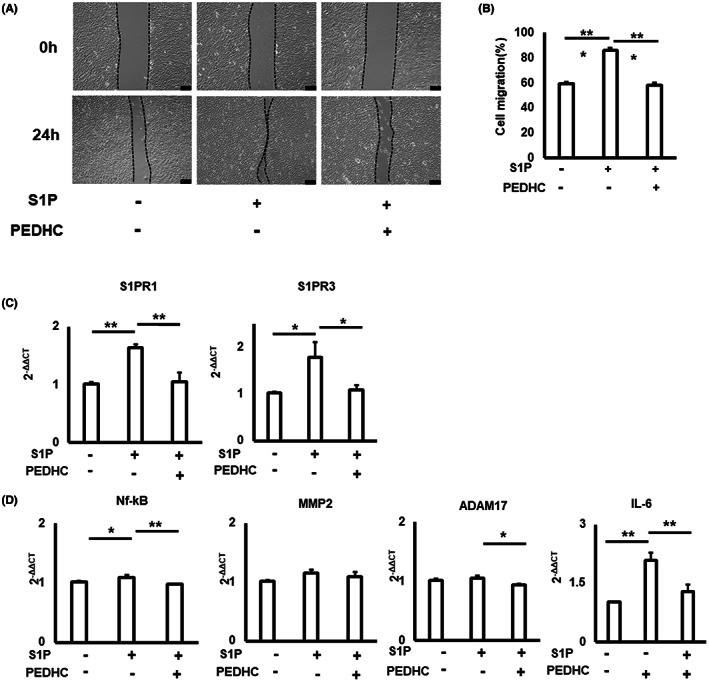
*Porphyromonas gingivalis*‐derived PEDHC inhibits S1P‐mediated migration of OECM‐1 cells. Representative images (A) and migration quantification (B) of oral squamous OECM‐1 cells at different time points exposed to S1P (1 μM) alone or in combination with PEDHC (1 μg/mL). Expression patterns of S1PR receptors, S1PR1 and S1PR3, (C) and genes associated with degradation of the basement membrane and extracellular matrix, including NF‐kB, MMP2, ADAM17, and IL‐6 (D) in OECM‐1 cells. Data are shown from three independent experiments. *p < 0.05, **p < 0.01, ***p < 0.001.

Finally, we tested the impact of PEDHC on the S1P‐mediated migration of OECM1 and the expression of S1PR1 and S1PR3 receptors. To our knowledge, migration of OECM‐1 cells significantly elevated in response to S1P (Figure 2A, B). In contrast, the S1P‐mediated migration of OECM‐1 was diminished in the presence of PEDHC in vitro. In addition, PEDHC downregulated the expression patterns of *NF‐kB*, and *IL6* mRNAs as well as *S1PR1* and *S1PR3* mRNAs in OECM‐1 cells elicited by S1P (Figure 2C, D; Figure S4B). Besides the positive pleiotropic impact of S1P on physiological chemoattraction, several studies also reported that the ligation of S1P to its S1PR1 and S1PR3 congenic receptors promotes chemotactic migration of cancer cells.^17^ More specifically, S1P/S1PR1 axis accelerates tumour progression by upregulating IL‐6.^22^ In addition, S1PR1 can also activate ERK to enhance cell survival and promote cell migration in fibrosarcoma and Hodgkin lymphoma.^23^ Furthermore, human breast cancers predominantly express S1PR3.^24^ Our data also agree with earlier published observations indicating that the bacterial‐derived lipids induce host immune responses and contribute to the S1P‐mediated signalling.^5^, ^25^

Collectively, these data indicated that PEDHC downregulates the expression of *ASAH1 mRNA* leading to the diminished survival of OECM‐1 cells via the accumulation of intracellular apoptotic ceramides in vitro. In addition, *P. gingivalis*‐derived PEDHC could also reduce the S1P‐mediated migration of OECM‐1, supporting our key finding that PEDHC plays a crucial inhibitory impact on the survival and migration of oral squamous carcinoma cells. To fully determine the role of PEDHC in cancer pathophysiology, further experimental steps should bring us closer to identifying and validating novel therapeutic targets and regimens for oral cancer. Most certainly, we will have gained a deeper insight into the relationships among oral bacterial virulence factors, host acid ceramidase and intracellular ceramides in the context of OSCC.

## AUTHOR CONTRIBUTIONS

**Chiaki Yamada:** Conceptualization (equal); data curation (equal); investigation (lead); methodology (equal); project administration (equal); writing – original draft (lead). **Anny Ho:** Data curation (equal); investigation (equal); methodology (equal); project administration (equal). **Amilia Nusbaum:** Formal analysis (equal); investigation (equal); methodology (equal). **Ruijuan Xu:** Data curation (equal); formal analysis (equal); methodology (equal). **Mary Ellen Davey:** Funding acquisition (equal); investigation (equal); methodology (equal); resources (equal); writing – original draft (equal). **Frank Nichols:** Data curation (equal); resources (equal); writing – original draft (equal). **Cungui Mao:** Data curation (equal); funding acquisition (equal); methodology (equal); writing – original draft (equal). **Alexandru Movila:** Conceptualization (equal); data curation (equal); formal analysis (lead); funding acquisition (lead); resources (lead); validation (lead); writing – original draft (equal).

## CONFLICT OF INTEREST STATEMENT

The authors have no conflicts of interest to declare.

## Supporting information


**Supplementary Figure 1.** *Porphyromonas gingivalis*‐derived phosphoethanolamine dihydroceramide (PEDHC) elevates total concentration of ceramide and dihydroceramide species in oral squamous OECM‐1 cells in vitro. Data are shown from four independent experiments. *p < 0.05, **p < 0.01, ***p < 0.001.
**Supplementary Figure 2.** Expression of different ceramidase genes in healthy OBA‐9 and oral squamous OECM‐1 cells in vitro. Acid ceramidase: ASAH1, neutral ceramidase; ASAH2, and alkaline ceramidase −1, −2, −3: ACER1, ACER2 and ACER3. Data are shown from four independent experiments. **p < 0.01, ***p < 0.001.
**Supplementary Figure 3.** Impact of acid ceramidase inhibitor, LCL521, on the proliferation of OECM‐1 squamous cells in vitro. (A) Expression of ASAH1 mRNA in OECM‐1 in the presence of LCL‐521 inhibitor. (B) Expression of genes associated with degradation of the basement membrane and extracellular matrix, including NF‐kB, MMP2, ADAM17 and IL‐6 in OECM‐1 cells. (C) Expression of S1P receptors, S1PR1 and S1PR3, in OECM‐1 cells exposed to LCL521. LCL‐521 inhibits proliferation (D) and migration (E, F) of OECM‐1 cells. Data are shown from four independent experiments. *p < 0.05, **p < 0.01, ***p < 0.001.
**Supplementary Figure 4.** Inhibitory effects of live wild‐type P. gingivalis W83 (P.g‐WT) and dihydroceramide sphingolipids null ΔPG1780 (P.g‐SphNull) strains (A) and PEDHC purified from P. gingivalis ATCC33227 strain (B) on the expression of ASAH1, S1PR1 and S1PR3 mRNAs in OECM‐1 cells in vitro. Data are shown from four independent experiments. *p < 0.05, **p < 0.01, ***p < 0.001.

## Data Availability

Data available on request from the authors.
