# Supplementary material for: Inhibitory effect of Porphyromonas gingivalis‐derived phosphoethanolamine dihydroceramide on acid ceramidase expression in oral squamous cells
Source: J Cell Mol Med. 2023 Apr 5;27(9):1290–5. doi: 10.1111/jcmm.17722 (PMC10148054; doi:10.1111/jcmm.17722)
Supplement: Supplementary file 1 — Supplementary Figure 1. Porphyromonas gingivalis‐derived phosphoethanolamine dihydroceramide (PEDHC) elevates total concentration of ceramide and dihydroceramide species in oral squamous OECM‐1 cells in vitro. Data are shown from four independent experiments. *p < 0.05, **p < 0.01, ***p < 0.001. Supplementary Figure 2. Expression of different ceramidase genes in healthy OBA‐9 and oral squamous OECM‐1 cells in vitro. Acid ceramidase: ASAH1, neutral ceramidase; ASAH2, and alkaline ceramidase −1, −2, −3: ACER1, ACER2 and ACER3. Data are shown from four independent experiments. **p < 0.01, ***p < 0.001. Supplementary Figure 3. Impact of acid ceramidase inhibitor, LCL521, on the proliferation of OECM‐1 squamous cells in vitro. (A) Expression of ASAH1 mRNA in OECM‐1 in the presence of LCL‐521 inhibitor. (B) Expression of genes associated with degradation of the basement membrane and extracellular matrix, including NF‐kB, MMP2, ADAM17 and IL‐6 in OECM‐1 cells. (C) Expression of S1P receptors, S1PR1 and S1PR3, in OECM‐1 cells exposed to LCL521. LCL‐521 inhibits proliferation (D) and migration (E, F) of OECM‐1 cells. Data are shown from four independent experiments. *p < 0.05, **p < 0.01, ***p < 0.001. Supplementary Figure 4. Inhibitory effects of live wild‐type P. gingivalis W83 (P.g‐WT) and dihydroceramide sphingolipids null ΔPG1780 (P.g‐SphNull) strains (A) and PEDHC purified from P. gingivalis ATCC33227 strain (B) on the expression of ASAH1, S1PR1 and S1PR3 mRNAs in OECM‐1 cells in vitro. Data are shown from four independent experiments. *p < 0.05, **p < 0.01, ***p < 0.001. [file JCMM-27-1290-s001.pdf]

Supplementary Material and Methods

Bacterial strain culture

Live wild-type *P. gingivalis* strain W83 (*P. gingivalis*-wild type), and a dihydroceramide sphingolipids-null mutant (W83 ΔPG1780) were used in this study. *P. gingivalis* W83 ΔPG1780 mutant strain was previously generated and characterized by Dr. Mary Ellen Davey group [1]. Both strains were grown on the brain heart infusion agar (BHI; BBL) supplemented with 5% sheep's blood, hemin, menadione, yeast extract (BD), cysteine, sodium bicarbonate, and sodium thioglycolate under anaerobic conditions at 37°C for 5 – 7 days. Bacteria were harvested in the mid- to late-exponential phase, and their concentration was measured using the Nanodrop (the O.D. at 600 nm corresponding to 10^8 cells/ml).

Purification of phosphoethanolamine dihydroceramide (PEDHC) from *Porphyromonas gingivalis*

Phosphoethanolamine dihydroceramide, PEDHC, was purified from *Porphyromonas gingivalis* (ATTC 33277) as previously described [2]. To stimulate human cells, PEDHC was diluted in phosphate-buffered saline (PBS) at a concentration of 1 mg/ml and then briefly sonicated (2 min, 3 W).

Cell lines and reagents

Human Oral Squamous Carcinoma OECM-1 cell line (Sigma-Aldrich) was cultured in RPMI-1640 medium (VWR), supplemented with 10% fetal bovine serum (Atlanta Biologicals), 2 mM L-glutamine, and 1% penicillin/streptomycin (Gibco). Altogether, 2×10^5 cells/well were seeded into a 12-well plate at 37 °C in 5% CO2 overnight and then stimulated with either live wild-type *P. gingivalis W83* or W83 ΔPG1780 (10^5 cells/ml or 10^6cells/ml) for 3 h. In addition, some groups of OECM-1 cells were also exposed to purified PEDHC, or commercially available sphingosine-1-phosphate (S1P, Sigma-Aldrich), or LCL521, an acid ceramidase inhibitor (MedChemExpress) for 24 h.

Cell Proliferation Assays

To examine the viability of oral squamous carcinoma OECM-1 cells, the WST-1 assay kit (Cayman Chemical) was employed according to the manufacturer's recommendations.

Cell scratch test for OECM-1 cell migration

The cells were seeded at 2×10^5 cells/well in a 12-well plate. After the cells had grown to 100% confluency, the cell layer was scratched with a 200µl sterile pipette tip across the monolayer cells and then washed twice with PBS. Finally, the medium was replaced with a fresh medium containing 10% FBS in the presence or absence of live wild-type *P. gingivalis* W83 *or* W83 ∆PG1780 strains, or various concentrations of PEDHC, S1P, or LCL-521. Images of the cells were captured at 0 and 24 h using an inverted EVOS microscope (ThermoFisher). Marked edges along each wound were used to measure cell migration by considering the horizontal distance between the initial scratch and the scratch following migration using the ImageJ software.

LC-MS/MS ceramide analysis

OECM-1 cells were seeded at 1×10^6 cells/well in a 6-well plate. After 12 h, the medium was replaced with a fresh medium containing 10% FBS in the presence or absence of various concentrations of PEDHC and incubated for an additional 24 h. Finally, cells were collected and snap-frozen after being washed with ice-cold 25 mM Tris-HCl buffer (pH 7.4) containing 150 mM NaCl. The total dihydroceramide and ceramide were measured in the Lipidomics Core Facility at Stony Brook University.

RNA Extraction and real-time PCR

The total RNA was isolated using PureLink™ RNA Mini Kit (Life Technologies) and subjected to reverse transcription with the Verso cDNA Synthesis Kit (Thermo Fisher Scientific) in the presence of random primers and oligo-dT following the manufacturer's recommendations. The expression of human ASAH1 (Hs00602774_m1), ASAH2 (Hs01015655_m1), ACER1 (Hs00370322_m1), ACER2 (Hs04996319_g1), ACER3 (Hs00924388_m1), S1PR1 (Hs00173499_m1), S1PR3 (Hs00245464_S1), IL6 (Hs00985639_m1), NF-κB (Hs01042010_m1), MMP2(Hs00234422_m1), and ADAM17 (Hs01041915_m1) genes were measured using TaqMan™ Fast Advanced PCR Master Mix (Applied Biosystems, Life Technologies). Data were analyzed using the $2^{-\Delta\Delta Ct}$ method normalized to GAPDH (Hs02786624_g1).

Statistical analysis

Data are displayed as mean ± SEM. Statistical significance was evaluated using either the T-test or a one-way ANOVA with a post-hoc Tukey test. A $p < 0.05$ was considered statistically significant. Data were analyzed using PAST 2.1 statistical software.

Supplementary Reference

1. Moye, Z.D., et al., *Synthesis of Sphingolipids Impacts Survival of Porphyromonas gingivalis and the Presentation of Surface Polysaccharides.* Front Microbiol, 2016. **7**: p. 1919.
2. Nichols, F.C., et al., *Phosphorylated dihydroceramides from common human bacteria are recovered in human tissues.* PLoS One, 2011. **6**(2): p. e16771.

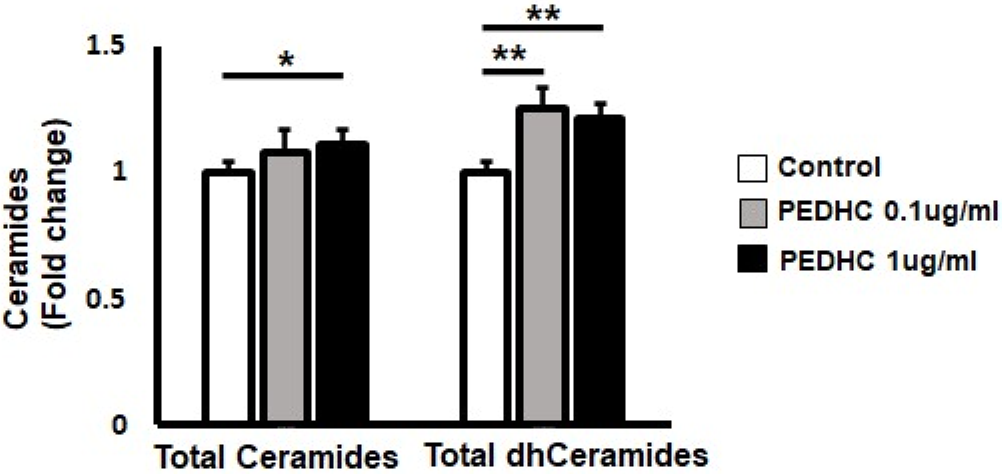

Supplementary Figure 1. *Porphyromonas gingivalis*-derived phosphoethanolamine dihydroceramide (PEDHC) elevates total concentration of ceramide and dihydroceramide species in squamous OECM-1 cells *in vitro*. N=4 *p <0.05, **p <0.01, *** p<0.001.

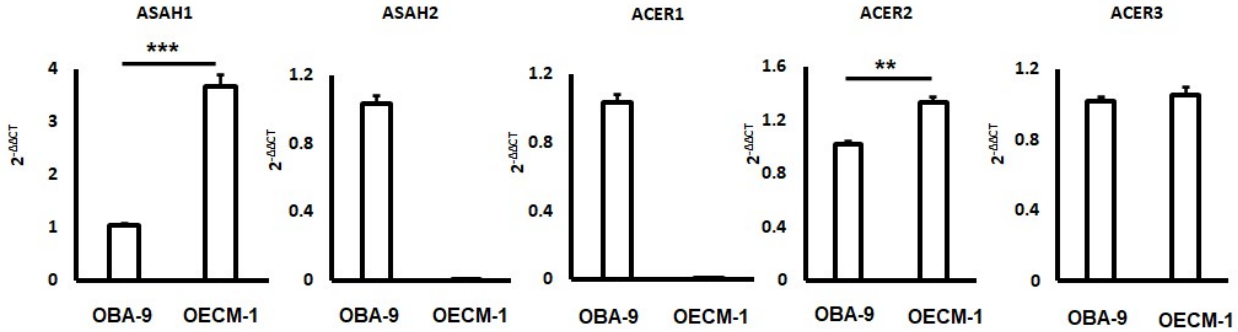

Supplementary Figure 2. Expression of different ceramidase genes in healthy OBA-9 and squamous OECM-1 cells *in vitro.* Acid ceramidase: ASAH1, neutral ceramidase; ASAH2, and alkaline ceramidase -1, -2, -3: ACER1, ACER2, and ACER3. N=3. ** *p* <0.01, *** *p* <0.001.

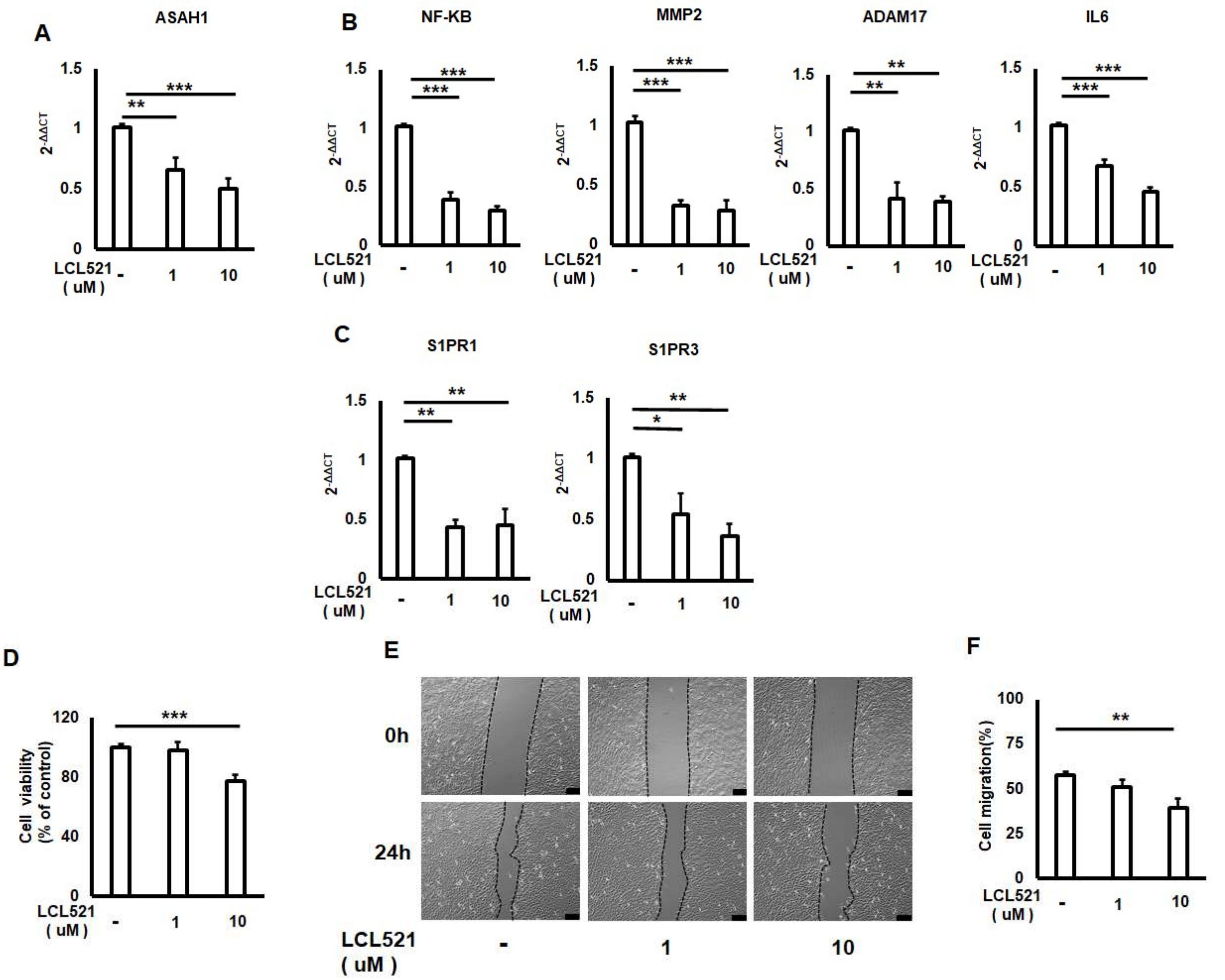

Supplementary Figure 3. Impact of acid ceramidase inhibitor, LCL521, on the proliferation of OECM-1 squamous cells *in vitro*. **A:** Expression of *ASAH1* mRNA in OECM-1 in the presence of LCL-521 inhibitor. **B:** Expression of genes associated with degradation of the basement membrane and extracellular matrix, including NF-kB, MMP2, ADAM17, and IL-6 in OECM-1 cells. **C:** Expression of S1P receptors, S1PR1 and S1PR3, in OECM-1 cells exposed to LCL521. LCL-521 inhibits proliferation **(D)** and migration **(E, F)** of OECM-1 cells. N=4 *p <0.05, **p <0.01, *** p<0.001.

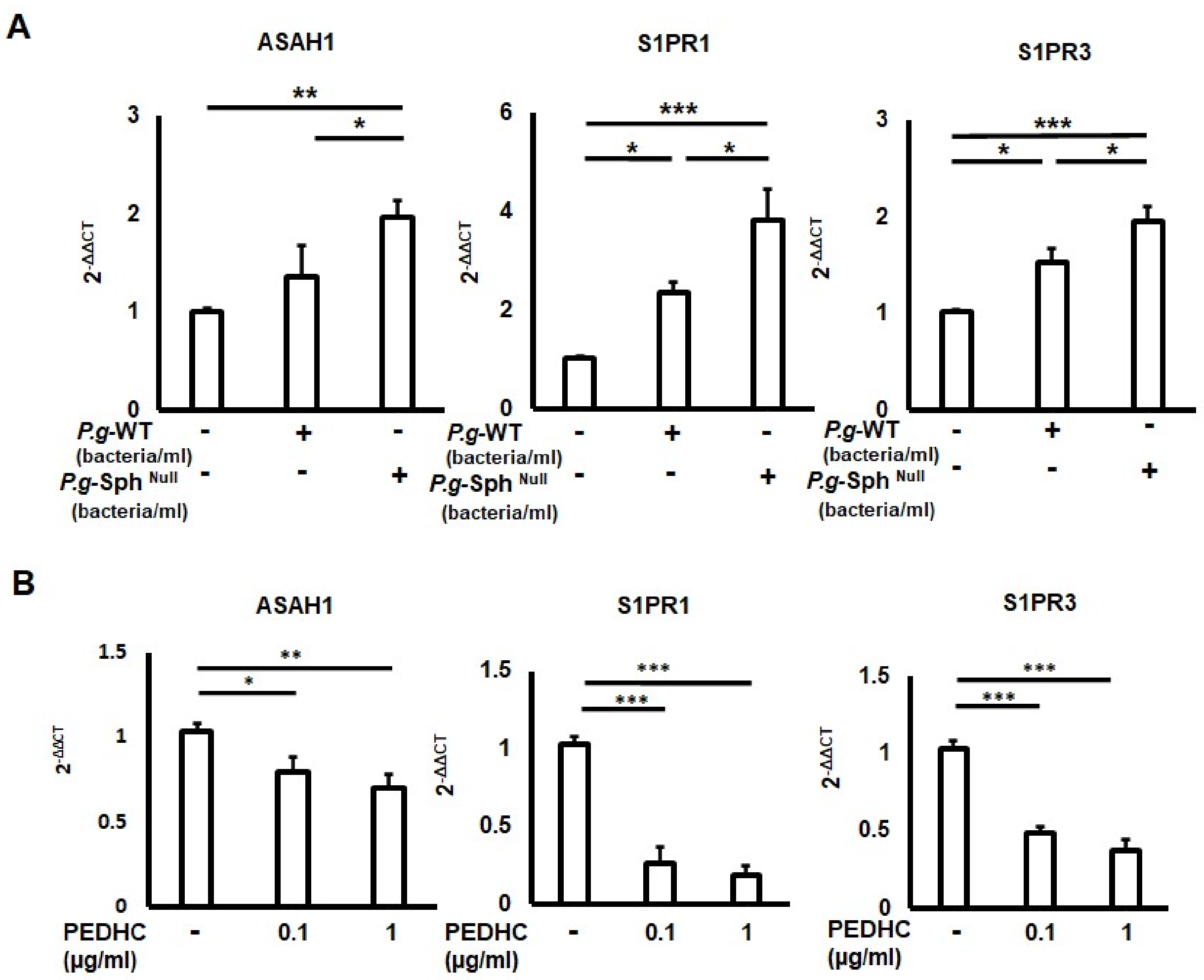

Supplementary Figure 4. Inhibitory effects of live wild-type *P. gingivalis* W83 (P.g-WT) and dihydroceramide sphingolipids null ∆PG1780 (*P.g*-Sph^{Null}) strains **(A)** and PEDHC, which was purified from wild-type *P. gingivalis* ATCC33227 strain **(B)** on the expression of *ASAH1*, *S1PR1*, and *S1PR3* mRNAs in OECM-1 cells *in vitro*. N=4 *p <0.05, **p <0.01, *** p<0.001.
